# Neutrophil-to-lymphocyte ratio as a sex-specific predictor of short-term mortality in hospitalised older adults with COVID-19: a pragmatic biomarker of inflammaging in acute vulnerability

**DOI:** 10.1186/s12979-025-00548-2

**Published:** 2025-12-29

**Authors:** Chiara Ceolin, Veronica Liberati, Vittorio Acunto, Margherita Vergadoro, Cristina Simonato, Sara Cazzavillan, Mario Virgilio Papa, Giulia Salerno Trapella, Bruno Micael Zanforlini, Chiara Curreri, Anna Bertocco, Giulia Gasparini, Maria Devita, Alessandra Coin, Paolo Simioni, Giuseppe Sergi, Marina De Rui

**Affiliations:** 1 Department of Medicine (DIMED), University of Padua, Padua, Italy; 2 Geriatrics Division, University Hospital of Padua, Padua, Italy; 3 Department of Neurobiology, Care Sciences and Society, Aging Research Center, Karolinska Institutet and Stockholm University, Stockholm, Sweden; 4 First Chair of Internal Medicine, Padova University Hospital, Padua, Italy; 5 School of Community Medicine and Primary Health Care, University of Padua, Padua, Italy; 6 Department of General Psychology (DPG), University of Padua, Padua, Italy; 7 First Chair of Internal Medicine & Thrombotic and Haemorrhagic Diseases Unit, Department of Medicine (DIMED), University of Padova, Padova, Italy

**Keywords:** COVID-19, Neutrophil-to-lymphocyte ratio, Mortality, Sex differences, Inflammaging, Immunosenescence

## Abstract

**Background:**

The neutrophil-to-lymphocyte ratio (NLR) is a low-cost inflammatory biomarker increasingly investigated in older populations as a cross-cutting indicator of immunosenescence and inflammaging. While elevated NLR has been shown to predict poor short-term outcomes in patients with COVID-19, its long-term prognostic role—particularly in older adults and in a sex-specific perspective—remains unclear. The aim of this study was to examine the association between admission NLR and 3.5-year all-cause mortality in older adults hospitalised with COVID-19, with a focus on sex-specific patterns.

**Methods:**

Prospective observational cohort study with 3.5-year follow-up. A total of 440 patients aged ≥ 65 years hospitalized with confirmed SARS-CoV-2 infection were enrolled. NLR was calculated at admission and dichotomized using the optimal cut-off value (12.63) identified via maximally selected rank statistics. Associations between NLR and mortality were assessed using Cox proportional hazards models, adjusted for age, sex, and vaccination status. Effect modification by sex was explored through interaction terms and sex-stratified analyses.

**Results:**

High NLR at hospital admission was independently associated with an increased risk of long-term all-cause mortality (adjusted HR 1.71; 95% CI: 1.21–2.43; *p* < 0.001). In sex-stratified analyses, this association remained significant only in females (HR 2.50; 95% CI: 1.49–4.22; *p* < 0.001). Sensitivity analyses revealed a significant association for mortality within 90 days of admission (HR 1.80; 95% CI: 1.15–2.81; *p* = 0.010), whereas no association was found for deaths occurring beyond 90 days (HR 0.83; 95% CI: 0.43–1.61; *p* = 0.58).

**Conclusions:**

NLR identifies a time-limited window of high vulnerability in older adults, particularly among women, reflecting the interplay between acute inflammation, immunosenescence, and processes typically associated with frailty. These findings highlight NLR as a pragmatic marker of inflammaging that could support sex-sensitive risk stratification and post-discharge interventions in geriatric care.

**Trial registration:**

Not applicable.

**Supplementary Information:**

The online version contains supplementary material available at 10.1186/s12979-025-00548-2.

## Key points

• The neutrophil-to-lymphocyte ratio (NLR) is a simple and low-cost inflammatory biomarker that predicts short-term mortality in older adults hospitalized with COVID-19.

• Elevated NLR at hospital admission was associated with increased 90-day mortality, but not with long-term (> 90 days) mortality risk.

• The prognostic value of NLR was particularly strong in women, suggesting sex-specific immune vulnerability in older patients.

• NLR may reflect acute inflammatory stress on a background of chronic immunosenescence and malnutrition, both common in older hospitalized populations.

• NLR could be easily implemented in routine geriatric assessment to support early risk stratification and post-discharge planning.

**Supplementary Information:**

The online version contains supplementary material available at 10.1186/s12979-025-00548-2.

## Background

Healthy ageing is increasingly understood through the lenses of immunosenescence and inflammaging—a chronic, low-grade inflammatory state that reduces immune reserve and amplifies vulnerability to acute stressors in late life [1]. This background inflammation contributes to the onset and worsening of geriatric syndromes and is associated with poor outcomes, including prolonged hospitalization, functional decline, and death [2]. Within this framework, the neutrophil-to-lymphocyte ratio (NLR) derived from a routine complete blood count has emerged as a pragmatic proxy of the balance between innate (neutrophil-driven) and adaptive (lymphocyte-driven) immunity, with prognostic value across conditions linked to ageing biology, including cancer, cardiovascular disease, sepsis, and geriatric syndromes [3–8]. NLR integrates neutrophil-dominant inflammatory activation and relative lymphopenia—hallmarks that map onto immunosenescence and may reflect diminished physiological reserve.

The SARS-CoV-2 (COVID-19) pandemic provided a natural stress test for such biomarkers. Patients with COVID-19 often exhibit a dysregulated immune response, marked by lymphocytopenia and functional exhaustion of key antiviral immune cells, such as natural killer (NK) cells and CD8 + T lymphocytes [4]. The degree of lymphocyte depletion has been shown to inversely correlate with disease severity [4, 9]. Elevated NLR has been consistently associated with disease severity, therapeutic response, and short-term mortality in hospitalized patients with SARS-CoV-2 infection [3, 5, 10–12].

However, two clinically relevant questions remain underexplored from an ageing-centred perspective. First, does NLR identify a time-limited window of heightened acute-to-early recovery phase that is actionable for prevention and care planning in older adults, rather than predicting distant outcomes indiscriminately? Second, are there sex-specific patterns consistent with known sexual dimorphism in immune ageing that could inform targeted strategies? While most studies have focused on short-term outcomes, evidence for the long-term prognostic role of NLR—particularly in older adults and in post-COVID cohorts—remains scarce. Although associations between NLR and long-term mortality have been reported in non-COVID populations [13], its utility as a marker of inflammaging and immune vulnerability in older survivors is poorly characterised. Addressing these gaps is essential to translate an accessible, low-cost biomarker into geriatric practice.

Beyond the acute infection, NLR also captures a background pro-inflammatory milieu—shaped by immunosenescence, malnutrition, and multimorbidity—that underpins frailty and reduced healthspan in older adults [4, 14–16]. Integrating NLR into routine admission assessments could therefore enable preventive, life-course–oriented care: prioritising nutritional and rehabilitation referrals, optimising medication regimens, delivering tailored vaccination counselling, and intensifying post-discharge follow-up during the high-risk post-acute period.

To address these gaps, we conducted a longitudinal study to investigate the association between admission NLR and 3.5-year all-cause mortality in older adults hospitalised with COVID-19, with a specific focus on sex-specific patterns that could inform personalised risk stratification and follow-up care.

## Materials and methods

### Study population

The characteristics and recruitment criteria of the study population have been previously reported in detail [17–20]. Briefly, this analysis included a consecutive cohort of Caucasian patients aged >65 years admitted to the Geriatrics Unit of the Azienda Ospedale – Università Padova with confirmed SARS-CoV-2 infection, regardless of the reason for hospitalization. Exclusion criteria included advanced dementia with inability to cooperate with clinical assessment, terminal illness with life expectancy < 1 month, active malignancy under treatment, immunosuppressive therapy (including high-dose corticosteroids or chemotherapy), and incomplete clinical or laboratory data at admission.

The study was conducted in accordance with Good Clinical Practice standards and the principles of the Declaration of Helsinki. The protocol was approved by the local ethics committee (Comitato Etico per la Sperimentazione Clinica della Provincia di Padova; protocol number 16412/AO/23), and written informed consent was obtained from all participants.

### Data collection

Detailed clinical, pharmacological, and functional characteristics of the study population have been previously reported [17–20]. Clinical and pharmacological data were retrospectively collected from medical records by experienced physicians. These included patients’ vaccination status, the type of vaccine received, and the total number of doses administered. Additional information was recorded regarding the severity of the infection, presence of pulmonary consolidations, need for oxygen therapy during hospitalization and at discharge, admissions to intensive care units, and the number and types of antibiotics, antivirals, and corticosteroids administered, including corticosteroid dosage. Comprehensive medication data, including the total number of drugs prescribed, were also gathered.

At the time of admission, blood samples were collected to evaluate complete blood count, liver and renal function, D-dimer, C-reactive protein (CRP), and lipid profile. All laboratory tests were conducted according to standardized protocols at the Laboratory Medicine Unit of the University Hospital of Padua. Complete blood counts were obtained using a routinely calibrated automated hematology analyzer certified for clinical use. The neutrophil-to-lymphocyte ratio (NLR) was subsequently calculated.

Follow-up for all-cause mortality was conducted 3.5 years after hospital discharge.

### Statistical analysis

The optimal cut-off point for the NLR was identified using maximally selected rank statistics via the *survminer* package in R. Based on this threshold, participants were categorized into high and low NLR groups. Continuous variables are presented as mean ± standard deviation (SD) or median with interquartile range (IQR), depending on distribution, which was assessed by visual inspection and the Shapiro–Wilk test. Categorical variables are summarized as counts and percentages. Group comparisons were conducted using independent-sample Student’s t-tests or Mann–Whitney U-tests for continuous variables, and Chi-square tests for categorical variables.

Time-to-event data were analyzed using Kaplan–Meier survival curves stratified by NLR group, with differences evaluated via the log-rank test. Cox proportional hazards regression models were used to estimate hazard ratios (HRs) and 95% confidence intervals (CIs) for the association between NLR (high vs. low) and all-cause mortality. Model 1 was unadjusted; Model 2 was adjusted for age and vaccination status (and for sex in the overall sample). Proportional hazards assumptions were assessed using Schoenfeld residuals. Stratified analyses by sex were performed, and effect modification was further tested by including an interaction term (NLR group × sex) in an extended Cox model.

A sensitivity analysis was conducted restricting the sample to patients who died within 90 days of admission to assess whether associations differed in the early phase of follow-up.

All analyses were performed in R version 4.5.0 (R Foundation for Statistical Computing, Vienna, Austria). Two-sided p-values < 0.05 were considered statistically significant.

## Results

### Baseline characteristics

A total of 440 patients were included in the analysis. Based on maximally selected rank statistics, the optimal NLR cutoff value was determined to be 12.63. Consequently, participants were divided in higher NLR group (17%) and lower NLR group (83%). The median age of the sample was 87 years (IQR: 81–92), and 49% were female. Compared to those with low NLR, patients in the high NLR group were older (median age 90 vs. 87 years, *p* < 0.001) and more frequently received ≥ 2 antibiotics during hospitalization (*p* = 0.002). They also had higher oxygen flow rates at discharge (mean 1.3 vs. 0.5 L/min, *p* = 0.009) and elevated levels of CRP, D-dimer, and creatinine (*p* < 0.001 for all). No significant differences were observed in sex distribution, cohabitation status, smoking history, or vaccination status between the NLR groups. Full baseline characteristics are reported in Table 1.

**Table 1:** Characteristics of the sample, in all sample and by Neutrophil-Lymphocyte Ratio (NLR) group

Variable	Overall *N* = 440	NLR group		*p*-value
		High *N* = 73	Low *N* = 367	
Age	87 (81, 92)	90 (85, 94)	87 (79, 91)	< 0.001
Sex, females	214 (49%)	31 (42%)	183 (50%)	0.25
Cohabitation status				0.82
Alone	65 (17%)	10 (18%)	55 (17%)	
With family	255 (68%)	38 (67%)	217 (69%)	
With no family members	40 (11%)	6 (11%)	34 (11%)	
Institutionalized	13 (3.5%)	3 (5.3%)	10 (3.2%)	
Smoking habits				0.43
Never	147 (67%)	18 (62%)	129 (68%)	
Active	16 (7.3%)	1 (3.4%)	15 (7.9%)	
Previous	55 (25%)	10 (34%)	45 (24%)	
Vaccination status (at least one dose)	297 (74%)	46 (74%)	251 (74%)	0.96
Clinical variables				
Pneumonia at admission	224 (58%)	44 (68%)	180 (56%)	0.083
O_2_ at Emergency Department discharge (L/min)	0 (0, 6)	2 (0, 6)	0 (0, 4)	0.20
Need for intensive care	16 (7.2%)	1 (4.0%)	15 (7.6%)	0.99
Total number of antibiotics during hospital stay				0.002
0	60 (26%)	2 (6.3%)	58 (29%)	
1	115 (49%)	15 (47%)	100 (50%)	
> 2	58 (24.9%)	15 (46.6%)	43 (21.5%)	
O_2_ at Hospital discharge (L/min)	0.9 ± 3.05	1.3 ± 3.0	0.5 ± 3.1	0.009
Cortisone dosage at discharge (mg)	13 (10, 25)	13 (8, 25)	13 (10, 25)	0.51
CIRS-CI	5.00 (3.00, 6.00)	5.00 (3.00, 6.00)	4.00 (3.00, 6.00)	0.26
Total n drugs at discharge	8.0 (6.0, 10.0)	8.5 (6.0, 12.0)	7.0 (5.0, 10.0)	0.048
*Biochemical data*				
NLR	4.4 (2.6, 7.8)	18.4 (14.7, 22.2)	3.6 (2.3, 5.7)	< 0.001
CRP (mg/dl)	24 (8, 59)	69 (28, 170)	19 (7, 47)	< 0.001
D-dimer (ug/L)	444 (251, 734)	637 (383, 1,745)	408 (241, 635)	< 0.001
Serum Creatinine (µmol/l)	72 (53, 101)	96 (64, 151)	70 (51, 91)	< 0.001

Missing data: Cohabitatation status (*n* = 67), Smoking habits (*n* = 222), Pneumonia at admission and O2 at Emergency Department discharge (*n* = 54), Emergency department (*n* = 218), Antibiotics (*n* = 207)

Numbers are expressed as mean ± standard deviation, median (interquartile range), or count (percentages), as appropriate

*Abbreviations*: *NLR* Neutrophil-to-Lymphocyte Ratio, *O*_*2*_ Oxygen, *CIRS-CI* Cumulative Illness Rating Scale – Comorbidity Index, *CRP* C-Reactive Protein

With regard to sex differences (Supplementary Table 1), women were significantly older than men (median 89 vs. 86 years, *p* < 0.001). Pneumonia at admission was more frequent in males (63% vs. 53%, *p* = 0.049), while other clinical characteristics—including oxygen requirement at ED discharge, ICU admission, corticosteroid dosage, and number of antibiotics—did not differ significantly. Biochemical markers such as NLR, CRP, and D-dimer were similar across sexes, although NLR tended to be higher in men (median 4.6 vs. 4.3, *p* = 0.071). The proportion of patients with high NLR was also comparable between sexes (19% in men vs. 14% in women, *p* = 0.25). Serum creatinine levels were significantly higher in males (median 82 vs. 63 µmol/L, *p* < 0.001).

### Survival analysis

**Fig. 1:**
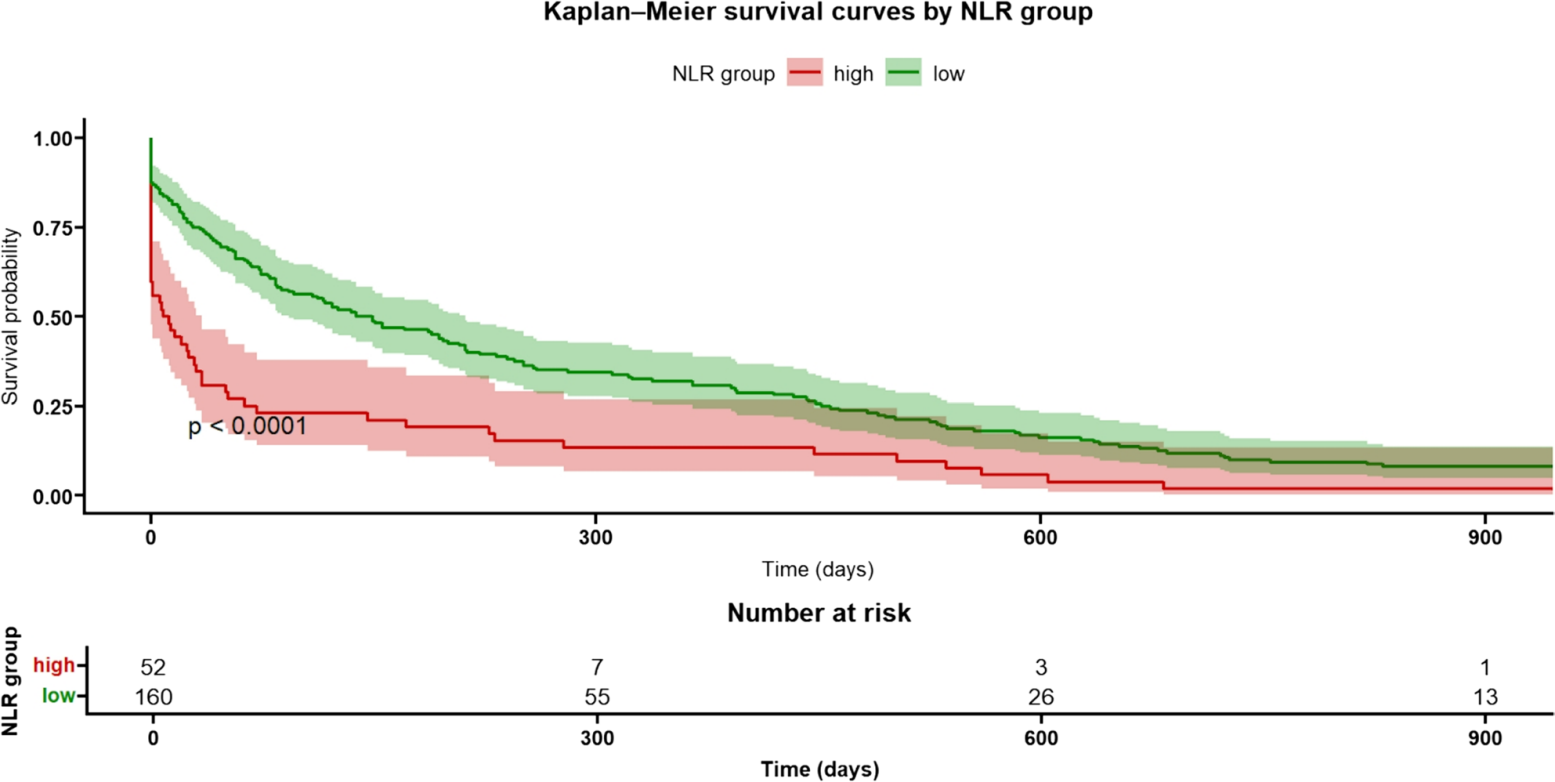
Kaplan–Meier survival curves by Neutrophil-lymphocyte ratio (NLR) group

**Fig. 2:**
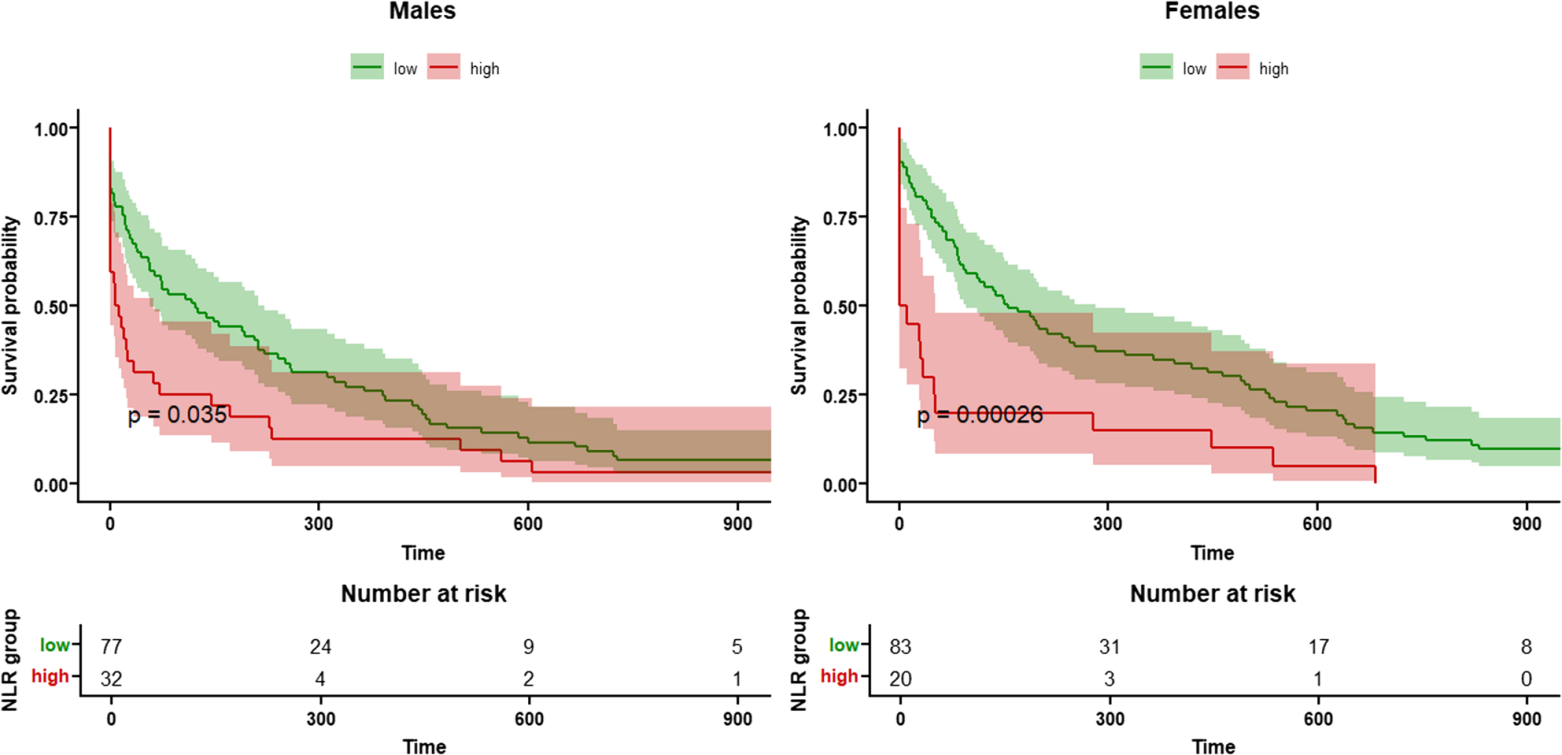
Kaplan–Meier survival curves stratified by sex and by Neutrophil-lymphocyte ratio (NLR) group

Over a maximum follow-up period of 3.5 years, a total of 246 deaths were recorded (55.9% of the cohort), with 120 (48.8%) occurring in men and 126 (51.2%) in women. Kaplan–Meier survival curves showed significantly lower survival among patients in the high NLR group (Fig. 1), with similar visual trends observed in both sexes (Fig. 2).

The results of the Cox proportional hazards models are presented in Table 2. In the unadjusted model (Model 1), high NLR was associated with increased risk of all-cause mortality in the overall sample (HR 1.95, 95% CI: 1.42–2.68, *p* < 0.001). This association remained significant in males (HR 1.59, 95% CI: 1.04–2.43, *p* = 0.031) and females (HR 2.59, 95% CI: 1.57–4.28, *p* < 0.001). After adjusting for age and vaccination status (Model 2), the association persisted in the overall sample (HR 1.71, 95% CI: 1.21–2.43, *p* < 0.001) and in females (HR 2.50, 95% CI: 1.49–4.22, *p* < 0.001), but was attenuated and no longer statistically significant in males (HR 1.34, 95% CI: 0.84–2.16, *p* = 0.223).

**Table 2:** Association between NLR (optimal cut-off) and all-cause mortality – Cox proportional hazards models

		All sample			Males			Females		
	Model	HR (NLR high vs. NLR low)	95% CI	p-value	HR (NLR high vs. NLR low)	95% CI	p-value	HR (NLR high vs. NLR low)	95% CI	p-value
Overall	Model 1	1.95	1.42–2.68	< 0.001	1.59	1.04–2.43	0.031	2.59	1.57–4.28	< 0.001
	Model 2	1.71	1.21–2.43	< 0.001	1.34	0.84–2.16	0.223	2.50	1.49–4.22	< 0.001

NLR was dichotomized based on the optimal cut-off identified via maximally selected rank statistics

*Abbreviations: HR* Hazard ratio *CI* Confidence Interval, *NLR* Neutrophil-lymphocyte ratio

Model 1: unadjusted

Model 2: adjusted for age, sex, and vaccination status

The sex-by-NLR interaction term added to the Cox model did not reach conventional significance (*p* = 0.075); however, it suggested a trend toward stronger associations between high NLR and mortality in females compared to males.

**Fig. 3:**
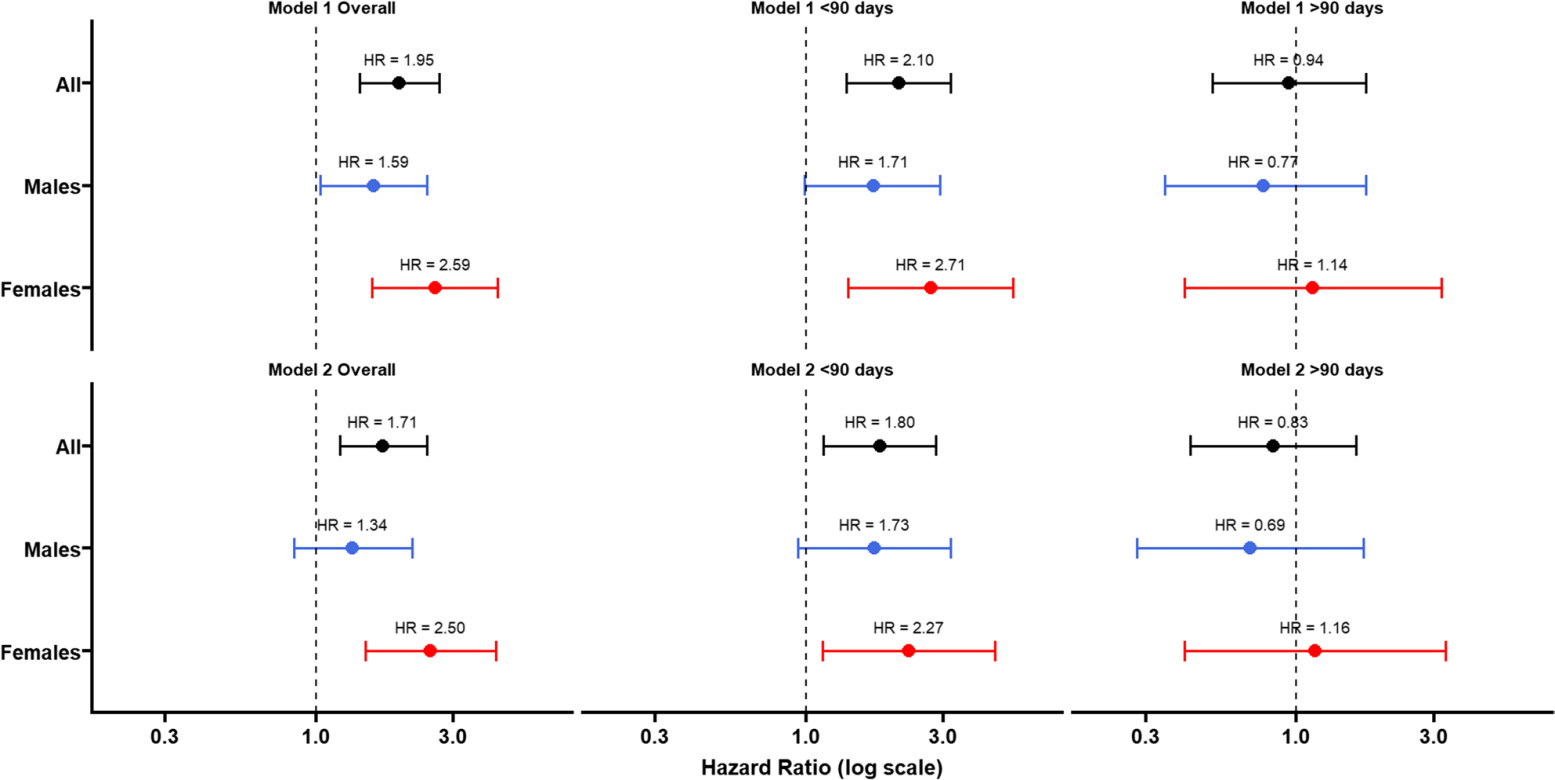
Hazard Ratios for High vs. Low NLR: Overall and Stratified Sensitivity Analyses (< 90 and > 90 Days). Notes: Model 1: unadjusted; Model 2: adjusted for age and vaccination status. Error bars represent 95% confidence intervals. Reference category: NLR low

A sensitivity analysis restricted to deaths occurring within 90 days of hospital admission confirmed the association between high NLR and increased mortality risk (HR = 1.80, 95% CI: 1.15–2.81, *p* = 0.010). However, when restricting the analysis to deaths occurring after 90 days, the association was no longer statistically significant (HR = 0.83, 95% CI: 0.43–1.61, *p* = 0.58) (Fig. 3).

In an exploratory analysis, we further distinguished between deaths occurring during hospitalization and those after discharge. A total of 27 deaths (6.1%) occurred during hospitalization and 219 after discharge. In-hospital mortality was significantly higher among participants with high NLR (20.5%) compared with those with low NLR (3.3%) (*p* < 0.001). When survival outcomes were categorized as alive, in-hospital death, or post-discharge death, the overall distribution significantly differed between NLR groups (*p* < 0.001). Given the limited number of in-hospital events, separate Cox regression analyses were not performed.

## Discussion

To our knowledge, this is the first study to investigate the association between the NLR and long-term mortality (3.5 years) in hospitalized older adults with COVID-19. Our central finding is that NLR does not predict mortality beyond 90 days, but rather identifies a time-limited window of acute vulnerability in older adults, consistent with concepts of immunosenescence and inflammaging exacerbated by acute stressors. This association was especially pronounced among women, suggesting that NLR may be a valuable tool for early mortality risk stratification in this high-risk population.

These results align with prior evidence from the broader COVID-19 literature, where elevated NLR values have consistently been associated with increased disease severity, poorer response to corticosteroid therapy, and higher short-term mortality [10–12, 21–24]. Most of these studies evaluated outcomes within 30 days of diagnosis or hospital admission [11, 12], while others extended follow-up to 60 days [10] or to the end of hospitalization [21–24]. By contrast, reports linking NLR to longer-term outcomes have generally involved younger or disease-specific cohorts—such as patients with cardiovascular disease, hematologic malignancies, or solid tumors—with follow-up periods ranging from one to ten years [13, 25–29]. In the present study, short-term mortality was defined as death occurring within 90 days after hospital admission, whereas long-term mortality included events up to 3.5 years of follow-up. This time frame situates our findings between the very early (30–60 day) outcomes explored in acute COVID-19 studies and the multi-year prognostic associations reported in chronic disease populations. Our results therefore support the view that, in older adults hospitalized for low-intensity COVID-19, elevated NLR primarily reflects acute inflammatory stress with immediate clinical impact, while its prognostic significance diminishes beyond the early post-acute phase. Supporting this interpretation, Galardo et al. [30] identified elevated NLR as a predictor of 30-day mortality in 953 older adults admitted for acute medical conditions, and Di Rosa et al. [31] similarly reported an association between higher NLR and in-hospital mortality in a large multicenter geriatric cohort.

Beyond acute infection, several factors may have contributed to higher NLR values at the time of hospitalization. In older adults, malnutrition, sarcopenia, multimorbidity, and polypharmacy interact with age-related inflammation and immune senescence, creating a background pro-inflammatory state that can amplify the acute response to infection [32]. Malnutrition and sarcopenia, in particular, are closely linked to chronic inflammation, muscle catabolism, and impaired immune competence [33]. Inadequate protein and energy intake, micronutrient deficiencies, and hormonal dysregulation can enhance neutrophil activation and reduce lymphocyte proliferation, thus increasing NLR [34]. Polypharmacy and long-term use of certain medications (e.g., corticosteroids, proton pump inhibitors) may further modulate leukocyte profiles [35, 36], while low vaccination uptake or ineffective immune memory can prolong the inflammatory burden [37]. Collectively, these interconnected mechanisms—encompassing nutritional status, muscle health, inflammation, and medication exposure—may explain the interindividual variability in NLR observed at hospital admission and highlight the contribution of chronic biological vulnerability to the acute inflammatory response.

Such background vulnerability may also help explain the temporal pattern of risk observed in our study, where NLR showed a stronger association with short-term than with long-term mortality. A possible explanation lies in the transient nature of systemic inflammation, which NLR captures. Elevated NLR may reflect an acute physiological stress response—due to infection, hospitalization, or multimorbidity—that increases immediate vulnerability. In contrast, long-term mortality in older adults is shaped by a complex interplay of frailty, chronic conditions, and socio-functional factors that are less directly influenced by inflammation at a single time point. Indeed, the prognostic value of NLR in older adults is likely rooted in its ability to reflect both inflammatory activation and immune status—two key elements in the pathophysiology of aging and acute illness.

Finally, to our knowledge, this is the first study to explore sex-specific differences in the association between NLR and long-term mortality in older adults hospitalized with COVID-19. While the overall predictive value of elevated NLR was confined to short-term mortality, our sex-stratified analyses revealed a significantly stronger association in females, both in the overall sample and particularly within the first 90 days post-admission. These findings are supported by growing evidence of sex-related differences in leukocyte profiles and immune-inflammatory responses. Several studies have documented higher neutrophil counts and NLR values in males compared to females, as well as across reproductive stages in women [38]. In particular, female-specific life events such as puberty, menopause, and reproductive history have been shown to affect white blood cell, neutrophil, and lymphocyte counts [39]. Around the age of 50, women typically experience a decrease in neutrophil percentage and an increase in lymphocyte percentage, leading to lower NLR values compared to premenopausal women—reflecting a distinct sexual dimorphism in immune cell composition [38]. This sexually dimorphic immune profile may partly explain why NLR carries greater prognostic value in older women. In the postmenopausal stage, shifts in immune function, hormonal status, and inflammatory regulation may amplify the vulnerability to acute inflammatory insults, rendering NLR a more sensitive marker of early mortality risk in this subgroup. In contrast, the weaker association observed in males could reflect a more stable pro-inflammatory state, less susceptible to short-term fluctuations in NLR.

Our findings indicate that NLR is not merely a predictor of outcomes in patients with COVID-19, but rather a simple and accessible proxy of the inflammatory and immune state that may reflect biological processes underlying frailty-related vulnerability in older adults. This interpretation is supported by our previous studies on the same cohort of older adults hospitalized for low-intensity COVID-19, which described nutritional decline, sarcopenia, and functional impairment as biological correlates of frailty [17–20]. This makes it a valuable tool to identify older adults who may benefit from early, personalized interventions, aligned with the promotion of healthy lifestyles, with the potential to reduce healthcare costs by limiting readmissions and prolonged hospital stays during the period of greatest vulnerability. Although our data derive from an acute infectious context, the NLR signal reflects cross-cutting mechanisms that can be leveraged beyond infection, acting as a clinical “red flag” to plan secondary and tertiary prevention strategies in other medical hospitalizations of older adults. Such pathways may include rapid nutritional assessment [40], prehabilitation [41], optimization of polypharmacy [42], counselling on vaccination and adherence [43], and intensified follow-up within the first 90 days—when vulnerability is highest. Furthermore, the stronger association observed in women paves the way for future research on sex-specific thresholds and the development of gender-responsive intervention bundles (e.g., more proactive nutritional screening, sarcopenia-focused rehabilitation, counselling on treatment adherence and vaccination).

### Limitations and strengths

Several limitations must be acknowledged. First, NLR was measured only once at admission; serial trajectories may provide greater insight into dynamic inflammatory changes. Second, we did not include standardized measures of frailty (e.g. CFS, ADL), which could confound associations between NLR and outcomes. Third, we did not account for competing risks of death from non-COVID causes, which should be addressed in future studies using Fine–Gray models. Moreover, information on the specific cause of death (COVID-19–related vs. non–COVID-19) was not systematically available from registry data. For this reason, we used all-cause mortality as a robust and unbiased outcome measure, while acknowledging that this may have limited our ability to distinguish deaths directly attributable to the acute infection from those related to post-COVID complications. In addition, we did not adjust for pneumonia at admission, as it was considered an intermediate variable on the causal pathway between systemic inflammation and mortality rather than an independent confounder. Finally, sex hormone levels were not available, which might have helped to explain observed sex-specific patterns.

Despite the limitations, strengths of our study include the relatively large cohort of very old patients (median age 87 years), the long follow-up, rigorous statistical analyses including sex-stratified and sensitivity models, and the pragmatic focus on a biomarker available worldwide at minimal cost.

## Conclusions

NLR at admission predicts early mortality (≤ 90 days), particularly in women, but not long-term outcomes. Its clinical utility therefore lies in identifying a short, actionable window of heightened vulnerability, rather than serving as a general predictor of late mortality.

## Supplementary Information

Supplementary Material 1.

## Data Availability

The datasets generated during and/or analysed during the current study are not publicly available due to privacy reasons but are available from the corresponding author on reasonable request.
