# Supplementary material for: Neutrophil-to-lymphocyte ratio as a sex-specific predictor of short-term mortality in hospitalised older adults with COVID-19: a pragmatic biomarker of inflammaging in acute vulnerability
Source: Immun Ageing. 2025 Dec 29;22:55. doi: 10.1186/s12979-025-00548-2 (PMC12750965; doi:10.1186/s12979-025-00548-2)
Supplement: Supplementary file 1 — Supplementary Material 1. [file 12979_2025_548_MOESM1_ESM.docx]

## *Supplementary Table 1.* Characteristics of the sample, by sex.

| **Variable** | **Males** N = 231 | **Females** N = 222 | **p-value** |
| --- | --- | --- | --- |
| **Age** | 86 (79, 91) | 89 (83, 94) | <0.001 |
| **Cohabitation status** |  |  | 0.066 |
| Alone | 31 (16%) | 34 (19%) |  |
| With family | 146 (74%) | 116 (63%) |  |
| With no family members | 14 (7.1%) | 27 (15%) |  |
| Institutionalized | 7 (3.5%) | 6 (3.3%) |  |
| **Smoking habits** |  |  | 0.024 |
| Never | 71 (61%) | 78 (76%) |  |
| Active | 8 (6.8%) | 8 (7.8%) |  |
| Previous | 38 (32%) | 17 (17%) |  |
| **Vaccination status (at least one dose)** | 164 (78%) | 144 (72%) | 0.15 |
| ***Clinical variables*** |  |  |  |
| **Pneumonia at admission** | 127 (63%) | 103 (53%) | 0.049 |
| **O2 at Emergency Department discharge (L/min)** | 0 (0, 6) | 0 (0, 6) | 0.96 |
| **Need for intensive care** | 11 (9.8%) | 5 (4.4%) | 0.11 |
| **Total number of antibiotics during hospital stay** |  |  | 0.34 |
| 0 | 30 (26%) | 31 (26%) |  |
| 1 | 51 (44%) | 66 (55%) |  |
| >2 | 35 (30.6%) | 23 (19.5%) |  |
| **O_2_ at Hospital discharge (L/min)** | 0.00 (0.00, 0.00) | 0.00 (0.00, 0.00) | 0.78 |
| **Cortisone dosage at discharge (mg)** | 13 (10, 25) | 13 (10, 25) | 0.68 |
| **CIRS-CI** | 5.00 (3.00, 6.00) | 4.00 (3.00, 6.00) | 0.035 |
| **Total n drugs at discharge** | 8.0 (6.0, 10.0) | 7.0 (5.0, 10.0) | 0.17 |
| *Biochemical data* |  |  |  |
| **NLR** | 4.6 (2.7, 9.0) | 4.3 (2.4, 7.0) | 0.071 |
| **NLR group** |  |  | 0.25 |
| high | 42 (19%) | 31 (14%) |  |
| low | 184 (81%) | 183 (86%) |  |
| **CRP (mg/dl)** | 26 (10, 60) | 23 (7, 58) | 0.44 |
| **D-dimer (ug/L)** | 444 (248, 682) | 453 (255, 797) | 0.52 |
| **Serum Creatinine (μmol/l)** | 82 (63, 118) | 63 (47, 84) | <0.001 |

Missing data: Cohabitatation status (n=67), Smoking habits (n=222), Pneumonia at admission and O2 at Emergency Department discharge (n=54), Emergency department (n=218), Antibiotics (n=207).

*Notes*: numbers are expressed as mean ± standard deviation, median (interquartile range), or count (percentages), as appropriate.

*Abbreviations*: NLR: Neutrophil-to-Lymphocyte Ratio; O_2_: Oxygen; CIRS-CI: Cumulative Illness Rating Scale – Comorbidity Index; CRP: C-Reactive Protein.
